# Dose Evaluation in 2-Phase Method for Advanced Esophageal Cancer by Hybrid Irradiation Techniques

**DOI:** 10.1016/j.ijpt.2024.100010

**Published:** 2024-04-25

**Authors:** Makoto Sasaki, Hiroyasu Tamamura, Yuji Tameshige, Yuya Azuma, Yoshikazu Maeda, Keiichiro Matsushita, Yoshitaka Sato, Shigeyuki Takamatsu, Kazuya Inoue, Yoji Tabata, Hitoshi Yoshimura, Kazutaka Yamamoto

**Affiliations:** 1 Proton Therapy Center, Fukui Prefectural Hospital, Fukui, Japan; 2 Kouseikai Proton Therapy Center, Nara, Japan; 3 Department of Radiology, Kanazawa University Graduate School of Medical Sciences, Ishikawa, Japan

**Keywords:** Two-phase method, Esophageal cancer, Proton broad-beam irradiation, VMAT, IMPT

## Abstract

**Purpose:**

In concurrent chemoradiotherapy for advanced esophageal cancer, a 2-phase method consisting of initial irradiation of a wide elective nodal region and boost irradiation of the primary lesion is commonly employed. Although dose escalation to the primary lesion may be required to achieve higher local control rates, the radiation dose to critical organs must not exceed dose constraints. To achieve an optimum balance of dose prescription and dose reduction to surrounding organs, such as the lungs and heart, we compared hybrid dose distributions and investigated the best combination of the following recent irradiation techniques: volumetric modulation arc therapy (VMAT), proton broad-beam irradiation, and intensity-modulated proton beam therapy (IMPT).

**Materials and Methods:**

Forty-five patients with advanced esophageal cancer whose primary lesions were located in the middle- or lower-thoracic region were studied. Radiotherapy plans for the initial and boost irradiation in the 2-phase method were calculated using VMAT, proton broad-beam irradiation, and IMPT calculation codes, and the dose-volume histogram indices of the lungs and heart for the accumulated plans were compared.

**Results:**

In plans using boost proton irradiation with a prescribed dose of 60 Gy(RBE), all dose-volume histogram indices were significantly below the tolerance limits. Initial and boost irradiation with VMAT resulted in the median dose of V_30 Gy(RBE)_(heart) of 27.4% and an achievement rate below the tolerance limit of 57.8% (26 cases). In simulations of dose escalation up to 70 Gy(RBE), initial and boost IMPT resulted in the highest achievement rate, satisfying all dose constraints in 95.6% (43 cases).

**Conclusion:**

Applying VMAT to both initial and boost irradiation is not recommended because of the increased risk of the cardiac dose exceeding the tolerance limit. IMPT may allow dose escalation of up to 70 Gy(RBE) without radiation risks to the lungs and heart in the treatment of advanced esophageal cancer.

## Introduction

Esophageal cancer is the eighth most common cancer worldwide and has a poor prognosis and high mortality rate, with East Asia having the highest mortality rate.^1^ Esophageal cancer is known to metastasize through the lymphatic drainage system, which is stretched in the superior-inferior (SI) direction, even at a relatively early stage.^2^, ^3^ Therefore, the therapeutic area that needs to be considered includes not only the recognizable tumor lesions and lymph node metastases but also the entire anatomical lymphatic system that the cancer infiltrates. From this perspective, a multidisciplinary treatment combining surgery, chemotherapy, and radiation therapy is often administered for advanced esophageal cancer. In Europe and the United States, while preoperative concurrent chemoradiotherapy (CCRT) and surgery are the standard treatment for advanced stage II and III esophageal cancer, CCRT is recommended as a curative treatment for patients who refuse surgery for unresectable tumors.^4^, ^5^ A randomized phase III intergroup 0123 study (Radiation Therapy Oncology Group 94-05) showed that a high radiation dose (64.8 Gy) did not increase the survival or local/regional control of esophageal cancer compared to the standard dose (50.4 Gy).^6^ Currently, chemotherapy (cisplatin 75 mg/m^2^ on day 1 and 5-fluorouracil 1000 mg/m^2^ on days 1-4) and radiation therapy (50.4 Gy) are commonly used as a standard regimen in CCRT for advanced esophageal cancer. However, the need for local control of over 50.4 Gy, especially in nonsurgery cases, is often reported,^7^, ^8^, ^9^ and the Japan Clinical Oncology Group 9516 trial showed that the CCRT regimen consisting of chemotherapy (cisplatin 70 mg/m^2^ on day 1 and 5-fluorouracil 700 mg/m^2^ on days 1-4) and radiation therapy (60 Gy) resulted in a complete response rate of 15% for stage IVa cases.^10^ In addition, recent improvements in local control techniques of radiotherapy have led some researchers to suggest the need for a prescription dose of 60 to 70 Gy.^11^, ^12^ In contrast, dose escalation increases the risk of late toxicities in surrounding organs at risk (OARs), including the lungs, heart, stomach, main bronchi, and spinal cord.^13^ As a CCRT irradiation method that preferentially satisfies the dose constraint of the spinal cord, applied techniques of three-dimensional conformal radiotherapy (3DCRT)—such as the 2-phase method and field-in-field method with 3 or 4 beams—have been established.^14^, ^15^, ^16^ The most standard CCRT irradiation technique is the 2-phase 3DCRT method, which consists of extensive “initial irradiation,” including elective nodal regions,^15^ and “boost irradiation” to the primary lesions and/or lymph node metastases. However, damage to OARs is inevitable for primary lesions in the middle-thoracic (Mt) and the lower-thoracic (Lt) region because it is difficult to formulate 3DCRT dose distributions that simultaneously satisfy dose constraints related to late toxicities in the spinal cord, lungs, and heart.^17^

To solve this problem, it is necessary to introduce radiation techniques with dose concentrations higher than those of 3DCRT. Owing to its physical characteristics, proton irradiation can deliver a sufficient dose to primary lesions while reducing the dose to the surrounding OARs.^18^, ^19^ However, the conventional irradiation system based on the proton broad-beam irradiation (PBB) was developed for local treatment within 150 to 200 mm^2^. Therefore, although it is suitable for “boost irradiation” to primary lesions, it is not originally appropriate as an irradiation method for a wide range of >300 mm square, such as an elective nodal region. Although a patch-field irradiation technique, in which multiple proton irradiation fields are connected,^20^ is often used for wide-range targets, there are many technical problems, such as the uncertainty of dose distribution at the joints of the irradiation fields, the response to internal fluctuations during isocenter movement, the limited irradiation arrangements, and the time and effort required for treatment. Therefore, for the “initial irradiation” of the 2-phase method for advanced esophageal cancer, it is desirable to have an irradiation technique that enables wide-range irradiation and high dose concentration. Recent radiation therapies that satisfy these requirements include intensity-modulated radiation therapy and volumetric modulation arc therapy (VMAT) using x-ray^21^, ^22^ or intensity-modulated proton beam therapy (IMPT) using a proton beam scanning method. In this study, we compared hybrid dose distributions using VMAT, PBB, and IMPT to determine the optimal combination of irradiation techniques in terms of target coverage and dose reduction for OARs, as well as the possibility of dose escalation to primary lesions to address clinical concerns.

## Materials and methods

### Patients

Among the patients treated with CCRT using x-rays and proton beams for esophageal cancer between November 2012 and July 2020 at our hospital, we focused on 45 patients in whom the primary lesion was located in the Mt or Lt region. In order to compare and examine cases with the same irradiation conditions, cases with lymph node metastases were excluded. All patient data were retrospectively extracted under an institutional review board-approved protocol. The sample size was determined so that the interval width at the 95% confidence level was <10% of the tolerance limit for the mean cardiac dose, using the population variance estimated from the 10 cases. The patients ranged in age from 50 to 86 years, with a median age of 67 years. The number of patients in stages I, II, III, and IV, according to the Union for International Cancer Control staging system, 8th edition,^23^ was 11, 11, 13, and 10, respectively. In all these cases, the primary lesion was in contact with a part of the heart.

### Radiation therapy planning system

In this study, the initial and boost radiation therapies were planned based on identical computed tomography (CT) image sets obtained using an Aquilion LB CT scanner (Canon Medical Systems, Inc, Tochigi, Japan). CT imaging was performed in 4-dimensional imaging mode, and the expiratory phase image sets were used as the planning image sets. VMAT, PBB, and IMPT planning were performed using the RayStation radiation therapy planning system (RaySearch Laboratories, Stockholm, Sweden), XiO-N proton therapy planning system (Mitsubishi Electric Corp, Tokyo, Japan), and Eclipse proton therapy planning system (Varian Medical Systems, Palo Alto, California), respectively. All the planning data recorded by these systems were transferred to the MIM Maestro image-processing tool (MIM Software Inc, Cleveland, Ohio), and the comprehensive plan was obtained by accumulating the initial and boost plan for each patient. Finally, we performed a dose-volume histogram (DVH) analysis on the accumulated plans.

### Contouring

The gross tumor volume (GTV) of the primary lesion was determined using the CT image set with reference to the generated positron emission tomography images and endoscopic clipping prior to the CT scan. The initial irradiation target for each case was the elective nodal region, which is a wide range spreading from the clavicular area with the supraclavicular lymph nodes to the abdominal area with the lymph nodes along the celiac artery,^15^, ^24^ regardless of the location of the GTV, and was assumed to be equal to the clinical target volume of the initial plan (CTV_initial_). The median length of the CTV_initial_ in the SI direction was 252 mm. The clinical target volume in the boost plan (CTV_boost_) included the GTV with a margin of 10 mm in the SI direction and 5 mm in other directions, excluding the overlapping bone and lung regions. The median length of CTV_boost_ in the SI direction was 90 mm. Since the setup margin included in the PTV margin reflects physical factors such as beam geometry, calculation code, radiation type, and drive precision of the equipment, the appropriate value for each irradiation device is usually used. However, in order to compare combinations of irradiation techniques in this study, the geometries of regions of interest should be standardized among VMAT, PBB, and IMPT planning. Therefore, the planning target volumes in the initial and boost plans (PTV_initial_ and PTV_boost_) included CTV_initial_ and CTV_boost_ with 5 mm margins including internal and setup margins. Furthermore, the spinal cord, lungs, and heart were contoured, used for DVH analysis, and confirmed by the doctors involved in this study.^25^ The dose to the heart was predicted to be highly dependent on the location of the primary lesion relative to the heart. Then, the lengths in the SI direction of the heart and the region that would overlap the heart and PTV_boost_ were defined as L_heart_ and L_overlap_, respectively, and the ratio of L_overlap_ to L_heart_ was used as an index of the location of the primary lesion relative to the heart.

### Dose prescription

To evaluate the biological effects of x-ray and proton beam irradiation, the doses in all radiation therapy plans were treated as biological doses instead of physical doses. The relative biological effectiveness of the proton beam was set to be 1.1 according to previous measurements,^26^ and the dose units were standardized to Gy(RBE).

Tamamura et al^27^ suggested that, for radiation therapy of over 60 Gy(RBE) administered for advanced esophageal cancer, a group that was administered a 36 Gy(RBE) dose to the elective nodal region had the same therapeutic effects and adverse events as that of a group administered a 40 Gy(RBE) dose. Therefore, in this study, the treatment planning policy was to prescribe 60 Gy(RBE) for primary lesions and 36 Gy(RBE) for the elective nodal region, excluding the primary lesions.

Since it was difficult to fairly evaluate PTV doses including physical factors among hybrid irradiation techniques, dose prescription conditions were set for CTV doses without physical factors. The mean doses of the CTV in the initial and boost plans (D_mean_[CTV_initial_] and D_mean_[CTV_boost_]) were set to 36 and 24 Gy(RBE), respectively, and the calculation conditions were determined such that doses of 95% of the CTV volume (D_95%_[CTV_initial_] and D_95%_[CTV_boost_]) were higher than 95% of the respective mean doses. The dose constraints for the OARs were set such that the maximum dose to the spinal cord (D_max_[spinal cord]) was <45 Gy(RBE), and the lung volume rate with a dose of over 5 Gy(RBE) (V_5 Gy(RBE)_[lungs]) was <50% in the accumulated plan. In the VMAT plan, because the above calculation conditions alone did not determine the tendency toward optimization, we added a condition where the dose gradient around the target was uniform by controlling the dose at points equidistant from the target.

### Calculation procedures

**Figure 1:**
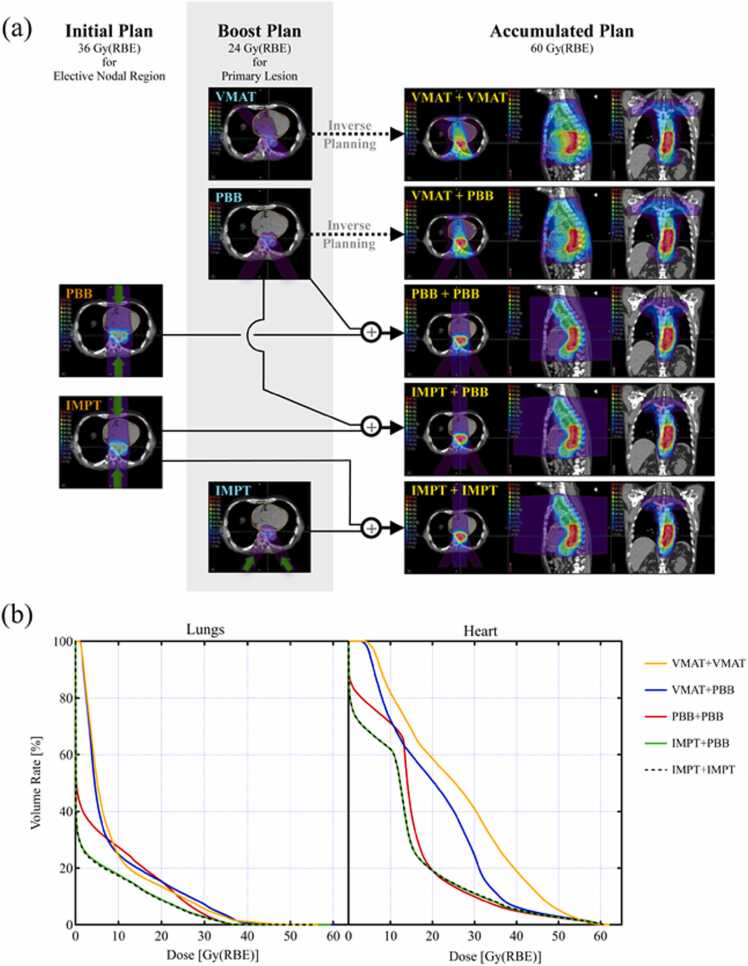
(a) Calculation procedure and (b) dose-volume histogram (DVH) curves of lungs and heart for a prescription dose of 60 Gy(RBE) for a typical case. (a) Plans for VMAT+VMAT and VMAT+PBB directly calculated the comprehensive dose based on the dose distributions of boost VMAT and boost PBB, respectively, using an inverse planning function of VMAT. Plans for PBB+PBB, IMPT+PBB, and IMPT+IMPT were obtained by simply accumulating the dose distributions for the initial and boost irradiations, calculated independently. (b) The orange, blue, red, and green solid lines denote DVH curves for the VMAT+VMAT, VMAT+PBB, PBB+PBB, and IMPT+PBB plans, respectively, and the black dashed line denotes the IMPT+IMPT plan. DVH curves of IMPT+PBB and IMPT+IMPT plans approximately overlap for the lungs and the heart. Abbreviations: IMPT, intensity-modulated proton beam therapy; PBB, proton broad-beam irradiation; and VMAT, volumetric modulation arc therapy.

The following 5 plans were calculated to investigate combinations of irradiation techniques for advanced esophageal cancer: (1) VMAT for both initial and boost irradiation (VMAT+VMAT), (2) VMAT for initial and PBB for boost (VMAT+PBB), (3) PBB for both initial and boost (PBB+PBB), (4) IMPT for initial and PBB for boost (IMPT+PBB), and (5) IMPT for both initial and boost (IMPT+IMPT). Figure 1a and b shows the calculation procedure and DVH curves, respectively, for a typical case. In the VMAT+VMAT and VMAT+PBB plans, the previously calculated dose data for boost VMAT and boost PBB, respectively, were imported into the VMAT treatment planning system as the basic dose, and the comprehensive dose distribution was calculated using the inverse planning function. To satisfy the dose constraints for the lungs, it was necessary to limit the x-ray irradiation angle range of 0° ± 65° and 180° ± 55° in almost all cases. In patients whose dose plans were difficult to satisfy the dose constraints of both the lungs and the spinal cord, priority was given to the dose of the spinal cord.

The dose distributions in the PBB+PBB, IMPT+PBB, and IMPT+IMPT plans, which consisted of proton beam irradiation only, were simple accumulations of the independently calculated initial and boost dose distributions. For the initial proton beam irradiation, a 2-field anterior-posterior and posterior-anterior beam arrangement was set at a dose-weight rate of 1:1. Because the target size exceeded the field aperture of 150 mm^2^, a patch-field irradiation technique was used in the initial PBB irradiation. In the boost proton beam irradiation, a 2-field left and right posterior oblique beam arrangement was used at a dose-weight rate of 1:1. One of the beams was designed at an irradiation angle such that the spinal cord was not within the field aperture. The irradiation angles of the 2 beams in the boost PBB and IMPT were set to be identical in each case. In the IMPT plan, a pseudoboard with a water-equivalent thickness of 50 mm was placed several tens of millimeters in front of the patient to control the dose with an appropriate proton spot size.

After calculating these plans for a prescription dose of 60 Gy(RBE) to the primary lesion according to the aforementioned procedure, we also simulated a dose escalation in which only boost dose fractions of 10 Gy(RBE) were added to all plans, resulting in a dose of up to 70 Gy(RBE) for the primary lesion.

### Evaluation indices

As evaluation indices, we derived DVH parameters such as V_10 Gy(RBE)_, V_20 Gy(RBE)_, V_30 Gy(RBE)_, V_40 Gy(RBE)_, and D_mean_ for the lungs and V_30 Gy(RBE)_ and D_mean_ for the heart, which are listed as tolerance limits in the National Comprehensive Cancer Network guidelines,^13^ and calculated the medians and 95% confidence intervals for these indices.

## Results

### DVH indices of lungs and heart in hybrid plans with a prescription dose of 60 Gy(RBE)

As shown in Table 1, the indices for the beam delivery of D_95%_(CTV_initial_), D_95%_(CTV_boost_), and D_max_(spinal cord) satisfied the original concept in all accumulated plans. Plans of VMAT+VMAT and VMAT+PBB accounted for 37.8% (17 cases) and 48.9% (22 cases) of all cases (45 cases), respectively, with V_5 Gy(RBE)_(lungs) exceeding 50% because of the priority of satisfying the dose constraint of the spinal cord, although the increase was <3%.

**Table 1:** Beam delivery indices.

Index (unit)	Concept		VMAT+VMAT	VMAT+PBB	PBB+PBB	IMPT+PBB	IMPT+IMPT
D_95%_(CTV_initial_) (GyE)	>34.2	Median	35.4	35.6	35.0	35.6	35.6
		95% CI	[35.3-35.6]	[35.4-36.0]	[34.8-35.2]	[35.6-35.7]	[35.6-35.6]
D_95%_(CTV_boost_) (Gy[RBE])	>57.0	Median	58.8	58.7	59.0	59.2	59.3
		95% CI	[58.7-58.9]	[58.7-58.8]	[58.8-59.1]	[59.1-59.3]	[59.3-59.4]
D_max_(spinal cord) (Gy[RBE])	<45.0	Median	40.7	42.0	33.6	33.2	29.7
		95% CI	[39.2-41.7]	[41.0-42.7]	[32.5-36.0]	[30.8-37.6]	[28.1-32.8]
V_5 Gy(RBE)_(lungs) (%)	<50.0	Median	49.7	50.0	28.3	19.4	16.7
		95% CI	[49.2-50.4]	[49.3-50.4]	[26.6-32.2]	[18.8-22.1]	[14.8-19.5]

**Abbreviations**: VMAT, volumetric modulation arc therapy; PBB, proton broad-beam irradiation; IMPT, intensity-modulated proton beam therapy; CTV_initial_, clinical target volume of the initial plan; CTV_boost_, clinical target volume in the boost plan; 95% CI, 95% confidence interval.

**Figure 2:**
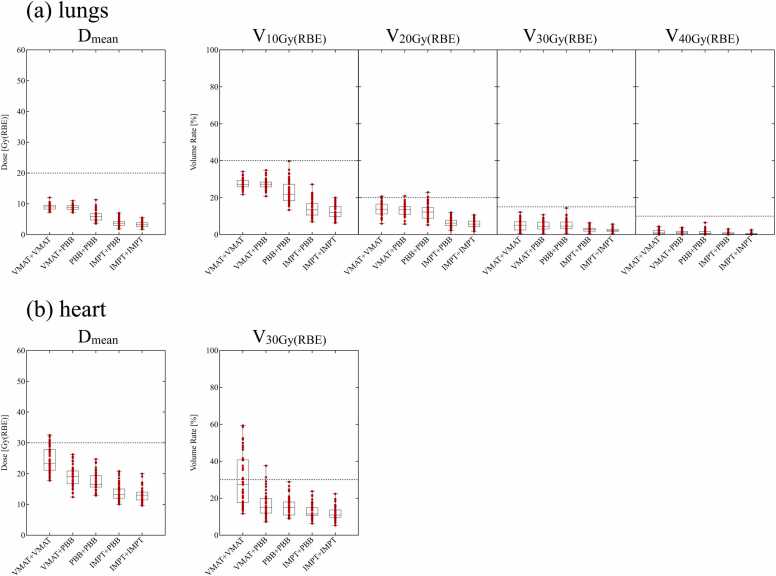
Dose-volume histogram indices of the (a) lungs and (b) heart for a prescription dose of 60 Gy(RBE). Horizontal dashed lines denote the tolerance limits.^13^ Abbreviations: IMPT, intensity-modulated proton beam therapy; PBB, proton broad-beam irradiation; and VMAT, volumetric modulation arc therapy.

**Figure 3:**
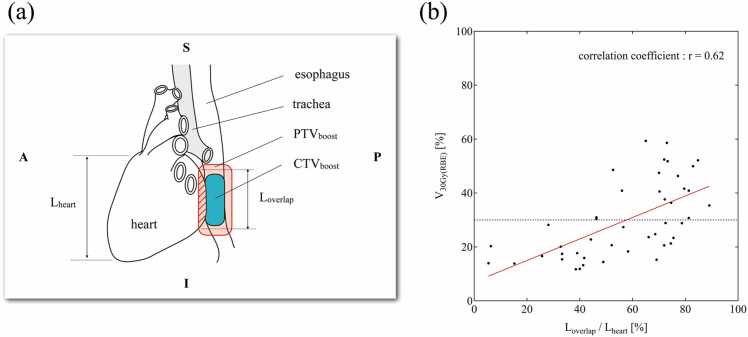
Correlation between V_30 Gy(RBE)_(heart) and the location of the heart and primary lesion. (a) Definition of L_overlap_ and L_heart_. (b) Scatter plot between V_30 Gy(RBE)_(heart) in the plan of initial and boost volumetric modulation arc therapy and L_overlap_/L_heart_. The horizontal dashed line denotes the tolerance limit of 30%.^13^ Abbreviations: CTV, clinical target volume; PTV, planning target volume.

The DVH indices for the lungs and heart at a prescribed dose of 60 Gy(RBE) are shown in Figure 2 and Table 2. All DVH indices for the lungs were sufficiently small compared to the tolerance limits. All DVH indices for the heart were significantly below the tolerance limits in all plans except for VMAT+VMAT; in the VMAT+VMAT plan, the median and achievement rate of dose constraint for V_30 Gy(RBE)_(heart) were 27.4% and 57.8% (26 cases), respectively. The V_30 Gy(RBE)_(heart) by VMAT+VMAT was widely dispersed across the tolerance limit, and we speculated that this was due to its dependence on the location of the primary lesion and heart. The scatter plot between V_30 Gy(RBE)_ and the ratio of L_overlap_ to L_heart_ is shown in Figure 3. The correlation coefficient was 0.62, suggesting a correlation between V_30 Gy(RBE)_ and L_overlap_/L_heart_.

**Table 2:** Dose-volume histogram indices of the lungs and heart for a prescription dose of 60 Gy(RBE).

Organ	Index (unit)	Tolerance limits^13^		Accumulated plan				
				VMAT+VMAT	VMAT+PBB	PBB+PBB	IMPT+PBB	IMPT+IMPT
Lungs	V_10 Gy(RBE)_ (%)	40.0	Median	27.1	27.3	21.9	13.4	12.0
			95% CI	[26.4-28.6]	[26.3-28.2]	[20.0-25.0]	[12.5-15.3]	[10.5-14.4]
	V_20 Gy(RBE)_ (%)	20.0	Median	13.8	13.6	12.2	6.2	5.7
			95% CI	[12.8-15.9]	[12.3-14.8]	[10.2-13.9]	[5.4-7.2]	[4.7-6.7]
	V_30 Gy(RBE)_ (%)	15.0	Median	5.1	4.5	4.6	2.8	2.3
			95% CI	[3.7-6.6]	[3.6-6.0]	[3.9-5.5]	[2.5-3.3]	[2.0-2.7]
	V_40 Gy(RBE)_ (%)	10.0	Median	0.9	1.0	0.7	0.4	0.3
			95% CI	[0.4-1.9]	[0.6-1.5]	[0.5-1.2]	[0.3-0.9]	[0.2-0.5]
	D_mean_ (Gy[RBE])	20.0	Median	9.0	8.8	5.9	3.7	3.2
			95% CI	[8.5-9.4]	[8.5-9.3]	[5.4-6.6]	[3.4-4.1]	[2.8-3.9]
Heart	V_30 Gy(RBE)_ (%)	30.0	Median	27.4	15.1	14.9	11.5	10.9
			95% CI	[20.6-36.4]	[12.6-18.1]	[13.4-17.3]	[11.1-13.9]	[10.1-12.9]
	D_mean_ (Gy[RBE])	30.0	Median	23.4	19.1	16.6	13.2	13.0
			95% CI	[22.3-26.9]	[17.6-20.6]	[16.2-18.5]	[12.6-14.8]	[11.9-13.8]

**Abbreviations**: VMAT, volumetric modulation arc therapy; PBB, proton broad-beam irradiation; IMPT, intensity-modulated proton beam therapy; 95% CI, 95% confidence interval.

### Dose escalation up to 70 Gy(RBE) by adding boost fractions

The DVH indices for dose escalation up to 70 Gy(RBE) with additional boost fractions are listed in Table 3. For the VMAT+VMAT and VMAT+PBB plans, it was already difficult to satisfy the irradiation conditions for both the spinal cord and lungs at the 60 Gy(RBE) prescription, and D_max_(spinal cord) values exceeded the tolerance limit in 68.9% (31 cases) and 46.7% (21 cases) of all cases for VMAT+VMAT and VMAT+PBB plans, respectively. All DVH indices in the PBB+PBB, IMPT+PBB, and IMPT+IMPT plans were significantly lower than the tolerance limits, and the achievement rates to satisfy all dose constraints were 77.8% (35 cases), 77.8% (35 cases), and 95.6% (43 cases), respectively.

**Table 3:** Dose-volume histogram indices of the lungs and heart for dose escalation up to 70 Gy(RBE).

Organ	Index (unit)	Tolerance limits^13^		Accumulated plan				
				VMAT+VMAT	VMAT+PBB	PBB+PBB	IMPT+PBB	IMPT+IMPT
Spinal cord	D_max_ (Gy[RBE])	45.0	Median	44.5	47.9	40.3	39.9	34.1
			95% CI	[43.5-46.2]	[46.6-48.3]	[38.1-42.4]	[37.2-42.7]	[32.7-37.1]
Lungs	V_5 Gy(RBE)_ (%)	50.0	Median	53.0	50.4	29.1	21.1	17.3
			95% CI	[52.6-53.9]	[50.0-51.4]	[27.4-33.3]	[19.8-23.0]	[15.4-20.0]
	V_10 Gy(RBE)_ (%)	40.0	Median	29.4	28.2	23.4	15.8	13.6
			95% CI	[28.4-30.7]	[26.9-29.0]	[21.7-26.6]	[15.1-18.2]	[12.0-16.3]
	V_20 Gy(RBE)_ (%)	20.0	Median	15.6	14.7	13.2	7.2	6.2
			95% CI	[14.2-17.6]	[13.3-16.2]	[11.3-14.4]	[6.1-8.3]	[5.2-7.1]
	V_30 Gy(RBE)_ (%)	15.0	Median	6.7	6.3	5.7	3.5	2.9
			95% CI	[5.2-8.3]	[5.3-7.3]	[4.8-6.7]	[2.9-4.1]	[2.5-3.2]
	V_40 Gy(RBE)_ (%)	10.0	Median	2.0	2.0	1.6	0.9	0.5
			95% CI	[1.1-3.4]	[1.3-2.8]	[1.1-2.1]	[0.6-1.5]	[0.3-0.8]
	D_mean_ (Gy[BE])	20.0	Median	9.8	9.3	6.3	4.3	3.4
			95% CI	[9.2-10.5]	[9.1-9.9]	[5.9-7.1]	[3.8-4.6]	[3.0-4.1]
Heart	V_30 Gy(RBE)_ (%)	30.0	Median	33.3	16.7	16.5	13.0	11.6
			95% CI	[29.5-47.5]	[15.1-21.4]	[14.7-18.8]	[12.4-15.5]	[11.0-14.3]
	D_mean_ (Gy[RBE])	30.0	Median	27.2	20.2	17.8	14.2	13.7
			95% CI	[24.9-30.6]	[18.6-21.5]	[16.9-20.2]	[13.6-6.2]	[12.4-14.7]

**Abbreviations**: VMAT, volumetric modulation arc therapy; PBB, proton broad-beam irradiation; IMPT, intensity-modulated proton beam therapy; 95% CI, 95% confidence interval.

## Discussion

The initial irradiation of the elective nodal region using a 2-phase method for advanced esophageal cancer requires both superior dose concentration and dose delivery over a wide range. As the PBB technique, with patch-field irradiation, has technical issues, alternative techniques such as VMAT or IMPT should be considered. Consequently, herein, we compared hybrid dose distributions using VMAT, PBB, and IMPT to determine the optimal combination of irradiation techniques in terms of target coverage and dose reduction for OARs, as well as the possibility of dose escalation to primary lesions as a clinical issue.

In the present study, no significant difference in V_30 Gy(RBE)_ for the lungs and heart was found between the VMAT+PBB and PBB+PBB plans; this suggests that dose concentrations above 30 Gy(RBE) surrounding the elective nodal region are not significantly different between VMAT and PBB. In contrast, because the dose distribution in the low-dose region below 30 Gy(RBE) is highly dependent on the passing volume of x-rays or proton beams, VMAT with a wide range of irradiation angles significantly increases the low-dose volume of the lungs and heart compared to that of PBB. However, these increases are sufficiently small relative to the tolerance limits. The substitution of PBB for VMAT during the initial irradiation should also be discussed, considering an optimum balance of treatment accuracy, operational efficiency, and dose distribution. IMPT using the inverse planning function generates a more conformal dose distribution surrounding the elective nodal region and causes dose reduction in the lungs and heart compared to PBB. Therefore, IMPT is the best alternative to PBB during initial irradiation. In actual proton scanning systems, the limitations of the scanning range and insertion of range shifters must be considered.^28^ Because IMPT should be actively used for wide-area irradiation, such as in elective nodal regions, future improvements in the technique are expected.

The DVH indices of the lungs and heart in all plans, except for VMAT+VMAT, were well below the tolerance limits in most cases, indicating significant contributions to cardiac dose reduction by proton boost irradiation (PBB or IMPT) for the treatment of esophageal cancer in the Mt or Lt region. In particular, the initial and boosted IMPT resulted in the most significant dose reduction to the lungs and heart. However, the low achievement rate of cardiac dose constraints and its correlation with the location of the primary lesion relative to the heart in the VMAT+VMAT plan suggest that VMAT+VMAT has a higher risk of irradiating the heart with a dose exceeding the tolerance limit as the area of contact between the primary lesion and the heart increases. Therefore, applying VMAT to the initial and boost irradiations is not recommended for esophageal cancer in the Mt or Lt region.

Hasatani et al^29^ suggested additional doses where residual lesions were observed on endoscopy at a prescription dose of approximately 50 Gy(RBE). Although determining the total dose in the latter part of the treatment period does not allow time for replanning, including CT imaging, calculations, and measurements for quality assurance, dose escalation by adding boost fractions would make it efficient to continue treatment. Because simulations of dose escalation from 60 Gy(RBE) to 70 Gy(RBE) showed that the IMPT+IMPT plan had extremely high achievement rates of lungs and cardiac dose constraints, it was inferred that the initial and boost IMPT allowed dose escalation by adding boost fractions. Thus, the use of IMPT in the 2-phase method for advanced esophageal cancer not only provides dose distribution with high dose concentration to targets and with superior dose reduction of OARs but also enables us to control an appropriate dose prescription according to residual lesions and radiation esophagitis during the treatment period.

Although this study focused on lung and heart doses, other OARs, such as the stomach, liver, and kidneys, for which tolerance limits have been listed in the NCCN guidelines, should also be evaluated. However, due to the limitations of this retrospective study, these organs as a whole were not necessarily included in the CT image sets, and a sufficient sample size could not be established. Since the location of the primary lesion relative to the heart contributes significantly to the heart dose, the stomach, liver, and kidney doses must also be systematically examined according to the location of the primary lesion. Further analysis, reconsidering a sufficient sample size for their evaluations, is crucial.

## Conclusion

In the 2-phase method for advanced esophageal cancer, the dose prescription of 60 Gy(RBE) to the primary lesion by the initial VMAT and boost PBB irradiations could be acceptable below the tolerance limits. Applying VMAT to both initial and boost irradiations increases the risk of irradiation to the heart above the tolerance limit, depending on the location of the primary lesion and heart, even with a dose prescription of 60 Gy(RBE) to the primary lesion. Therefore, it should not be recommended for esophageal cancer in the Mt or Lt regions. The use of IMPT for both initial and boost irradiations is considered the best irradiation technique for this treatment planning simulation and is expected to allow dose escalation from 60 to 70 Gy(RBE) according to the patient’s condition.

## Ethics

All patient data were collected using an internal review board-approved protocol.

## Funding

The authors received no funding for this study.

## Author contribution

Makoto Sasaki: Conceptualization, Methodology, Formal analysis, Data curation, Investigation, Writing- Original draft preparation. Hiroyasu Tamamura: Methodology, Validation, Formal analysis, Data curation, Writing- Review and editing. Yuji Tameshige: Methodology, Investigation. Yuya Azuma: Methodology, Investigation. Yoshikazu Maeda: Investigation, Writing- Review and editing. Keiichiro Matsushita: Investigation, Writing- Review and editing. Yoshitaka Sato: Investigation, Writing- Review and editing. Shigeyuki Takamatsu: Methodology, Validation, Writing- Review and editing, Supervision. Kazuya Inoue: Investigation. Yoji Tabata: Investigation. Hitoshi Yoshimura: Supervision. Kazutaka Yamamoto: Validation, Writing- Review and editing, Supervision. All authors have read and approved the final version of the manuscript.

## Declaration of Conflicts of Interest

The authors declare that they have no known competing financial interests or personal relationships that could have appeared to influence the work reported in this paper.
